# Correction: A large language model framework for sample-free population synthesis

**DOI:** 10.1371/journal.pone.0356492

**Published:** 2026-08-19

**Authors:** Michael Jones, Richard Dawson, Jon Mills

In Fig 10, the published figure shows results from a preliminary experiment instead of results from 20 repeated runs of the Newcastle case study. Please see the correct Fig 10 here.

**Fig 10:**
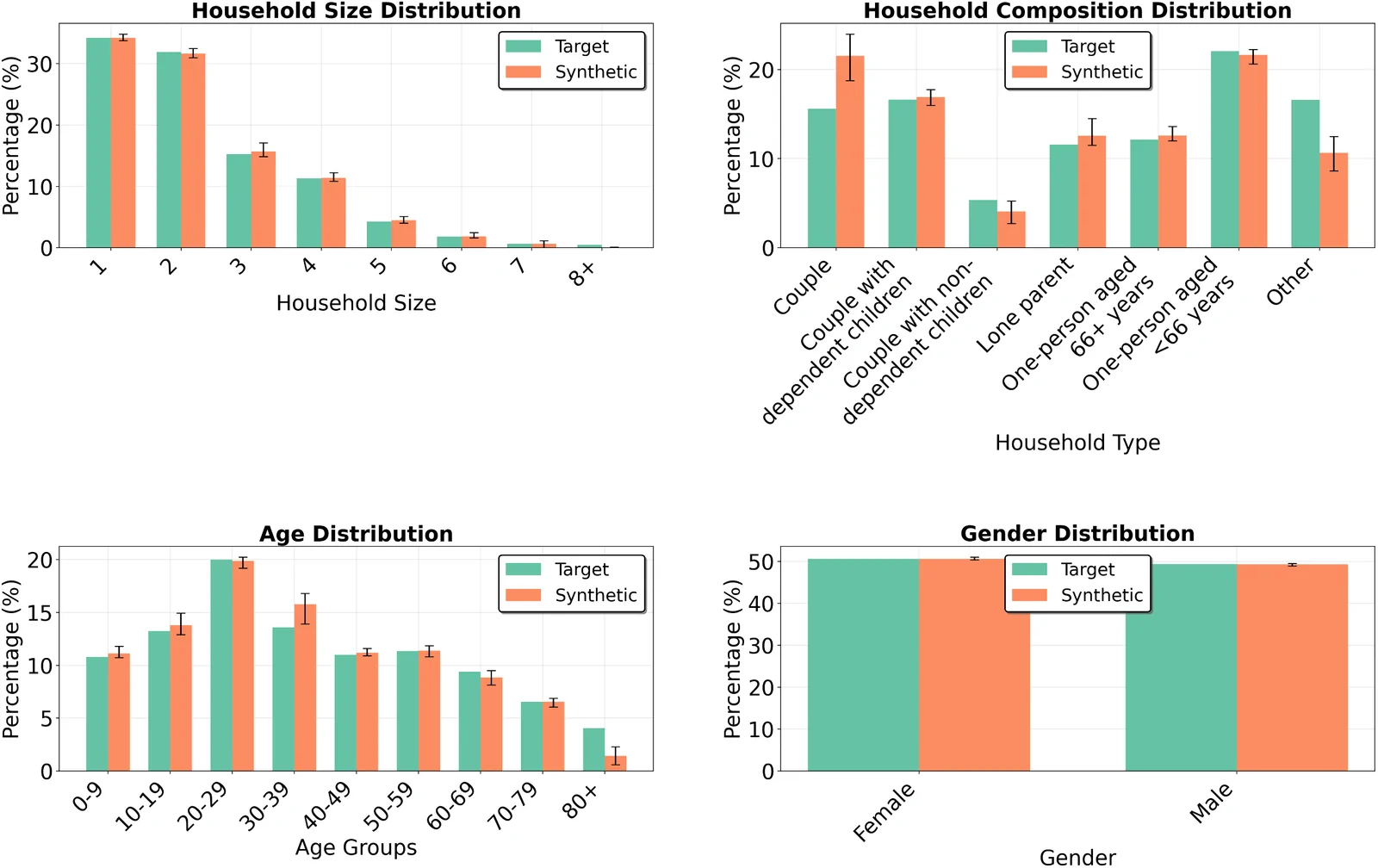
Comparison of target and synthetic marginal distributions across 20 independent runs (note different Y axis scales).
