# Supplementary material for: A large language model framework for sample-free population synthesis
Source: PLoS One. 2026 Jun 2;21(6):e0341704. doi: 10.1371/journal.pone.0341704 (PMC13229344; doi:10.1371/journal.pone.0341704)
Supplement: S1 File — Appendix A: example prompt, including distributional feedback data. Appendix B: preliminary experiment evaluating LLM querying without iterative distributional feedback. Appendix C: benchmarking results for all evaluated models, including age-gender population pyramids and distributional fit metrics. Appendix D: global case study results, including age-gender population pyramids and a comparison of SRMSE and Wasserstein metrics. Appendix E: scatter plots for univariate OLS regression analyses of country-level SRMSE predictors. Appendix F: meta-prompt used to generate prompt variants for sensitivity analysis. (DOCX) [file pone.0341704.s001.docx]

# Appendix A

TASK:

You are an AI assistant generating a realistic synthetic population for Newcastle upon Tyne, UK.

Your goal is to add one new household to a growing dataset, so that the generated population matches target distributions.

INSTRUCTIONS:

1. Review the current and target distributions for household size, composition, age, and gender (provided below).

2. Select a household size category where the current count is below its target.

3. Choose a valid household composition for the selected size, prioritizing categories that are currently under-represented.

- 1-person: contains a single adult of any age.

- 2-person: can be either a couple, a lone parent with a child, or unrelated housemates.

- 3+ person: can be a family (couples or lone parents with children, extended family), or unrelated housemates.

- Non-dependent children are aged 18 or older.

4. Select individuals for the household, prioritizing under-represented age groups and genders.

5. For each person, include:

- age (0–120)

- gender ("Male" or "Female")

- relationship (first must be "Head" and be an adult; valid values: "Spouse", "Partner", "Child", "Parent", "Sibling", "Grandchild", "Grandparent", "Housemate", "Lodger", "Aunt", "Uncle", "Nephew", "Niece", "Cousin", "Child-in-law", "Parent-in-law", "Sibling-in-law")

6. Output only a JSON object with a "household" array containing these individuals. Do not include markdown, formatting, or explanations.

DATA:

Household Size Distribution:

- 1: current = 40.0%, target = 34.2% (over-represented)

- 2: current = 43.3%, target = 31.9% (over-represented)

- 3: current = 10.0%, target = 15.3% (under-represented)

- 4: current = 6.7%, target = 11.3% (under-represented)

- 5: current = 0.0%, target = 4.3% (under-represented)

- 6: current = 0.0%, target = 1.8% (under-represented)

- 7: current = 0.0%, target = 0.7% (under-represented)

- 8+: current = 0.0%, target = 0.5% (under-represented)

Household Composition Distribution:

- One-person aged <66 years: current = 33.3%, target = 22.1% (over-represented)

- One-person aged 66+ years: current = 6.7%, target = 12.1% (under-represented)

- Lone parent: current = 13.3%, target = 11.6% (over-represented)

- Couple: current = 33.3%, target = 15.6% (over-represented)

- Couple with dependent children: current = 13.3%, target = 16.6% (under-represented)

- Couple with non-dependent children: current = 0.0%, target = 5.3% (under-represented)

- Other: current = 0.0%, target = 16.6% (under-represented)

Age Distribution:

- 0-9: current = 10.9%, target = 10.8% (over-represented)

- 10-19: current = 9.1%, target = 13.2% (under-represented)

- 20-29: current = 25.5%, target = 20.0% (over-represented)

- 30-39: current = 9.1%, target = 13.6% (under-represented)

- 40-49: current = 16.4%, target = 11.0% (over-represented)

- 50-59: current = 21.8%, target = 11.4% (over-represented)

- 60-69: current = 5.5%, target = 9.4% (under-represented)

- 70-79: current = 1.8%, target = 6.5% (under-represented)

- 80+: current = 0.0%, target = 4.1% (under-represented)

Gender Distribution:

- Male: current = 56.4%, target = 49.4% (over-represented)

- Female: current = 43.6%, target = 50.6% (under-represented)

GENERATED HOUSEHOLD:

# Appendix B

Prior to developing the framework described in Section 3, a preliminary experiment was conducted to evaluate whether repeated LLM querying, without feedback about the growing population, could produce a demographically representative synthetic population. The prompt supplied to the model contained task instructions, the output schema, and the target distributions, but omitted the empirical distributions of the households generated so far.

Distributional fit was poor (Figure B1). Household composition was dominated by "couple with dependent children" households, which accounted for 60% of generated records despite representing only 17% of the target. Similar patterns were evident elsewhere: 3- and 4-person households were overrepresented, and the age distribution was skewed towards adults aged 30–50 with children under 20. These results reflect the model's pre-training prior rather than the specified targets, confirming that supplying target distributions within the prompt is insufficient on its own to ensure they are reproduced. This motivated the iterative feedback mechanism described in Section 3.4.


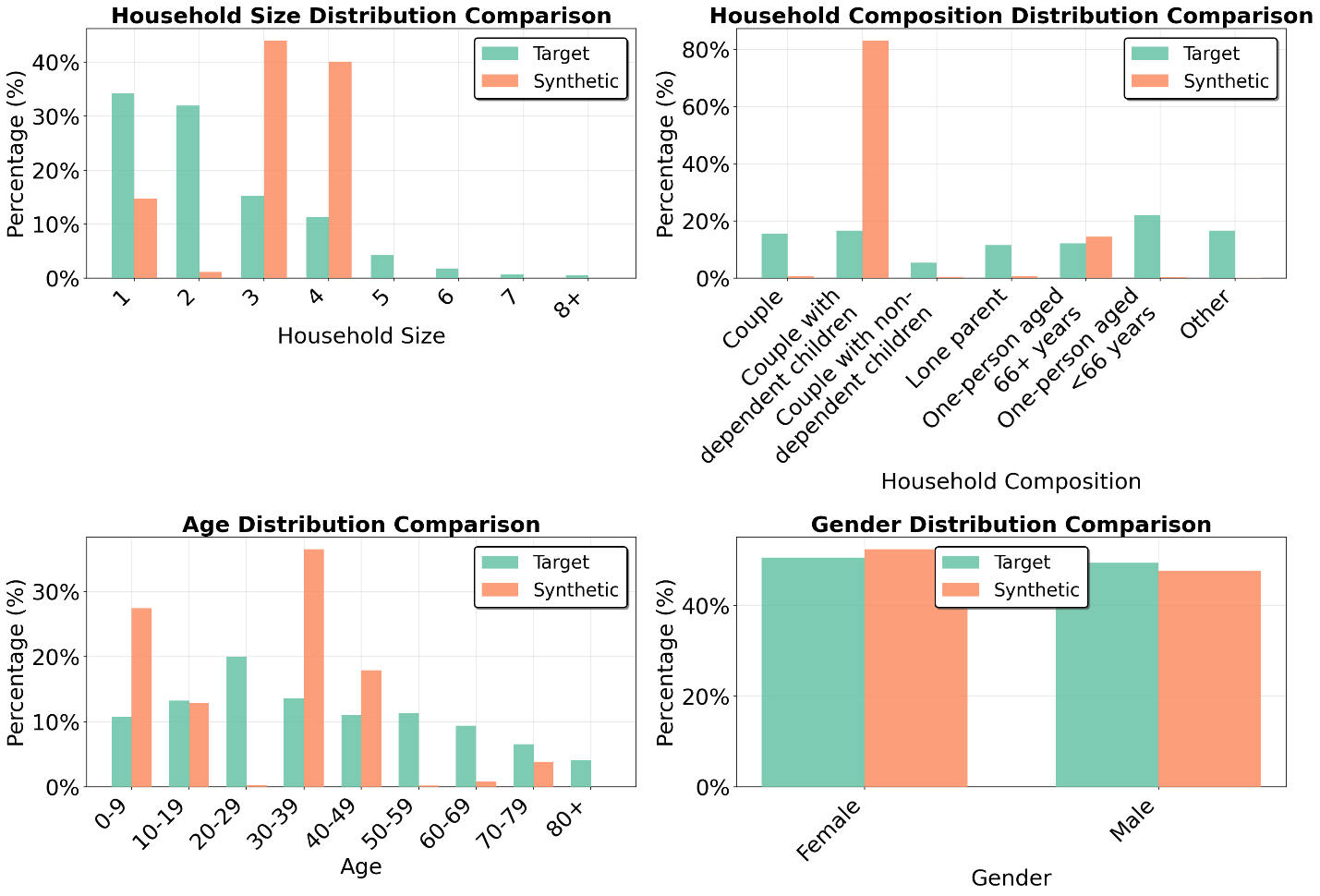


**Fig. B1** Marginal distributions produced without distributional feedback, compared against Newcastle upon Tyne targets.

# Appendix C
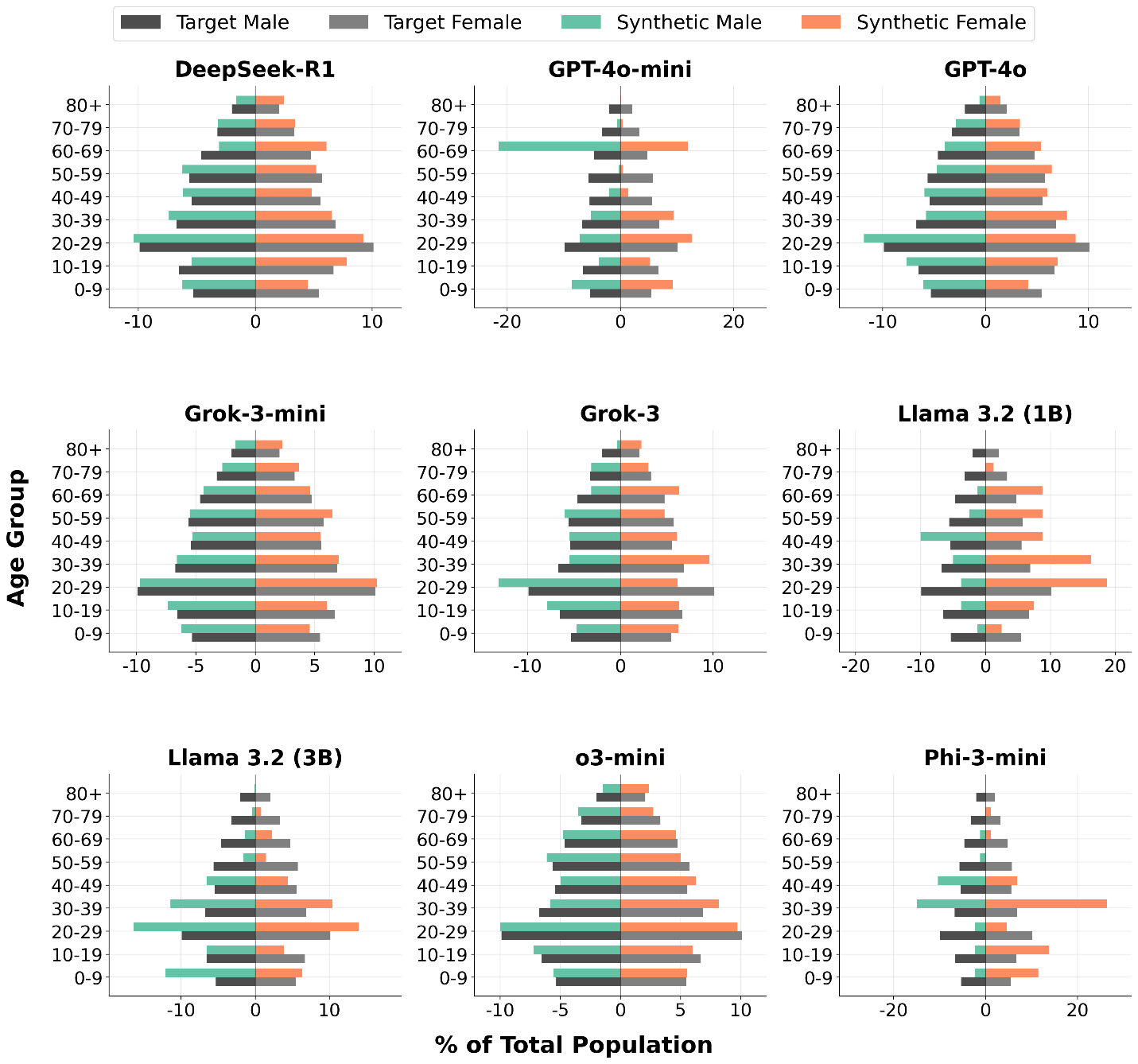


**Fig. C1** Age-gender population pyramids for all benchmarked models, based on an 800-household test case.

**Table C1** Distributional fit metrics for all benchmarked models across four demographic variables. JSD=Jensen-Shannon Divergence; SRMSE = Standardised Root Mean Square Error; $W_{1}$= Wasserstein Distance. $W_{1}$is reported for ordinal variables only (household size and age). Lower values indicate a better fit in all cases.

|  | **Household size** | | | **Household composition** | | **Age** | | | **Gender** | | **Mean** | | |
| --- | --- | --- | --- | --- | --- | --- | --- | --- | --- | --- | --- | --- | --- |
| **model** | **JSD** | **SRMSE** | $W_{1}$ | **JSD** | **SRMSE** | **JSD** | **SRMSE** | $W_{1}$ | **JSD** | **SRMSE** | **JSD** | **SRMSE** | $W_{1}$ |
| gpt-4o | 0.04 | 0.02 | 0.03 | 0.11 | 0.30 | 0.04 | 0.08 | 0.14 | 0.00 | 0.01 | 0.05 | 0.10 | 0.08 |
| gpt-4o-mini | 0.23 | 0.75 | 0.48 | 0.34 | 1.10 | 0.32 | 0.88 | 0.48 | 0.00 | 0.01 | 0.22 | 0.68 | 0.48 |
| deepseek-r1-0528 | 0.02 | 0.03 | 0.03 | 0.01 | 0.02 | 0.00 | 0.02 | 0.01 | 0.00 | 0.01 | 0.01 | 0.02 | 0.02 |
| o3-mini | 0.01 | 0.02 | 0.02 | 0.01 | 0.02 | 0.01 | 0.02 | 0.03 | 0.00 | 0.00 | 0.01 | 0.02 | 0.02 |
| grok-3 | 0.05 | 0.03 | 0.03 | 0.06 | 0.20 | 0.04 | 0.08 | 0.11 | 0.00 | 0.00 | 0.04 | 0.08 | 0.07 |
| grok-3-mini | 0.02 | 0.03 | 0.02 | 0.01 | 0.03 | 0.01 | 0.02 | 0.01 | 0.00 | 0.00 | 0.01 | 0.02 | 0.02 |
| llama3.2-1b | 0.54 | 1.46 | 2.77 | 0.54 | 1.84 | 0.21 | 0.45 | 0.55 | 0.16 | 0.44 | 0.36 | 1.05 | 1.66 |
| llama3.2-3b | 0.48 | 1.59 | 1.30 | 0.48 | 1.29 | 0.24 | 0.59 | 1.03 | 0.05 | 0.14 | 0.31 | 0.90 | 1.16 |
| phi3-mini | 0.44 | 1.74 | 0.90 | 0.44 | 1.23 | 0.34 | 1.04 | 0.93 | 0.11 | 0.30 | 0.33 | 1.08 | 0.92 |

# Appendix D
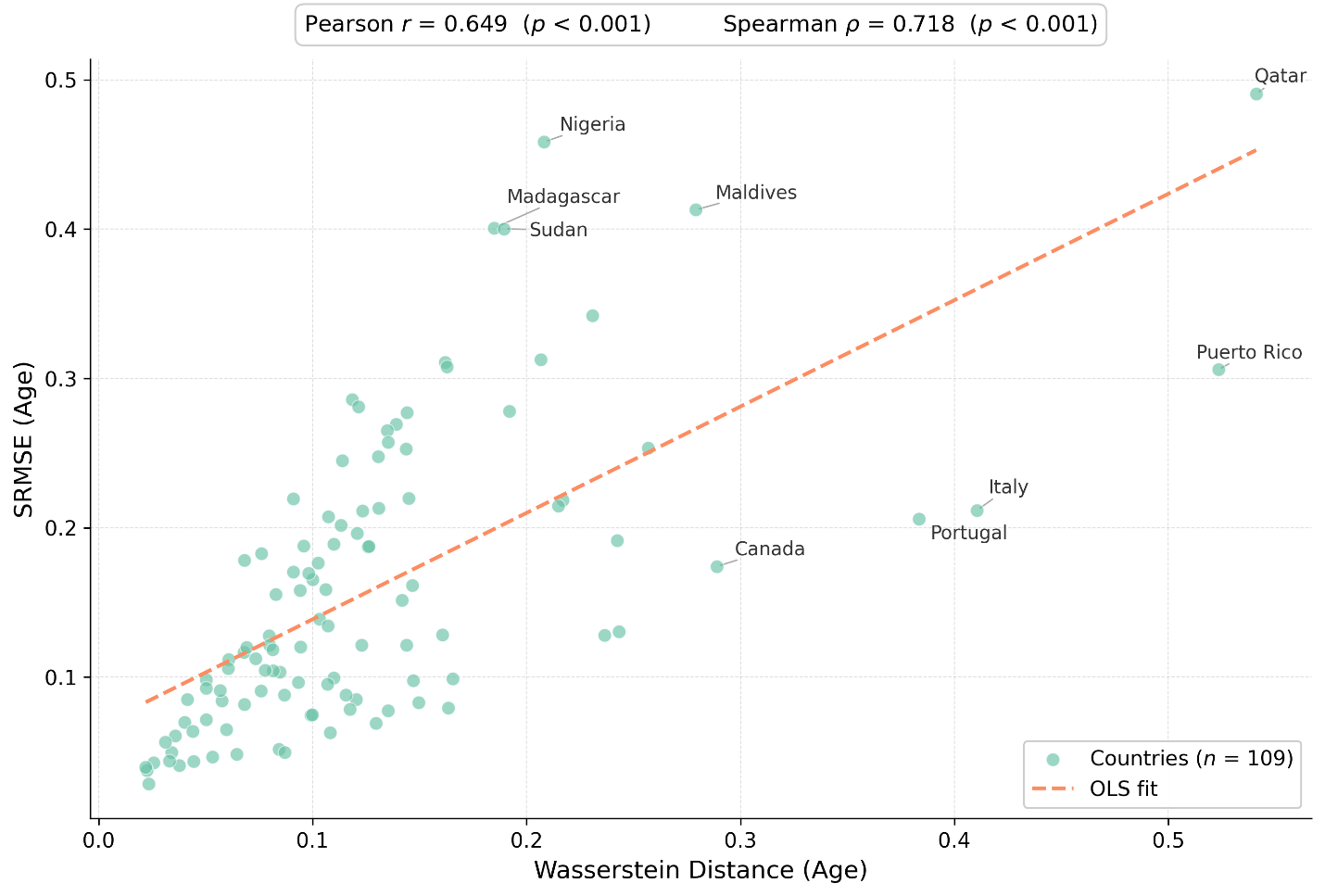
***Fig. D1*** *SRMSE versus Wasserstein distance for the age distribution across 109 countries with OLS fit shown.*



***Fig. D2*** *Synthetic and target age-gender population pyramids for countries A-D*

#
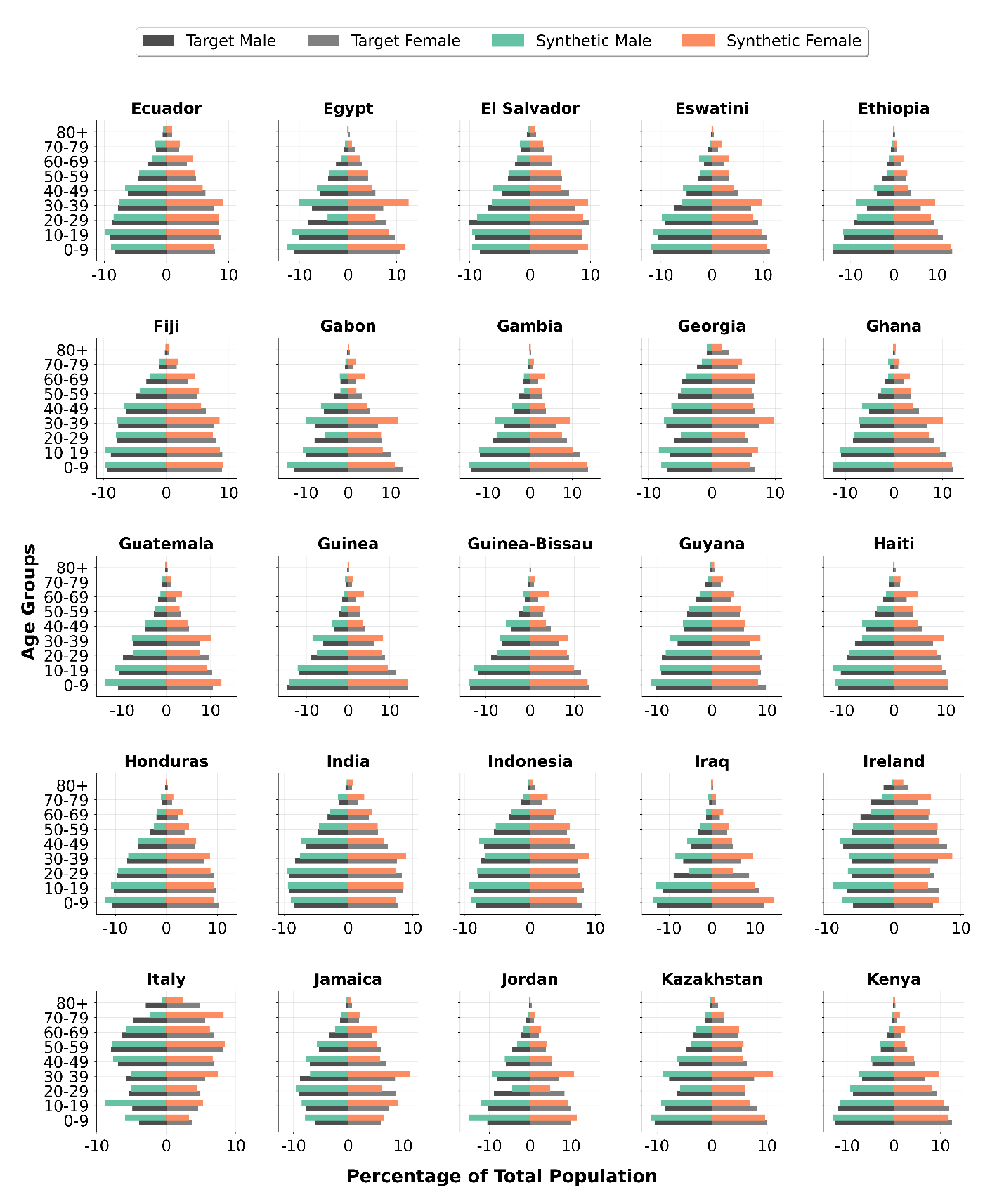
***Fig. D3*** *Synthetic and target age-gender population pyramids for countries E-K*

#
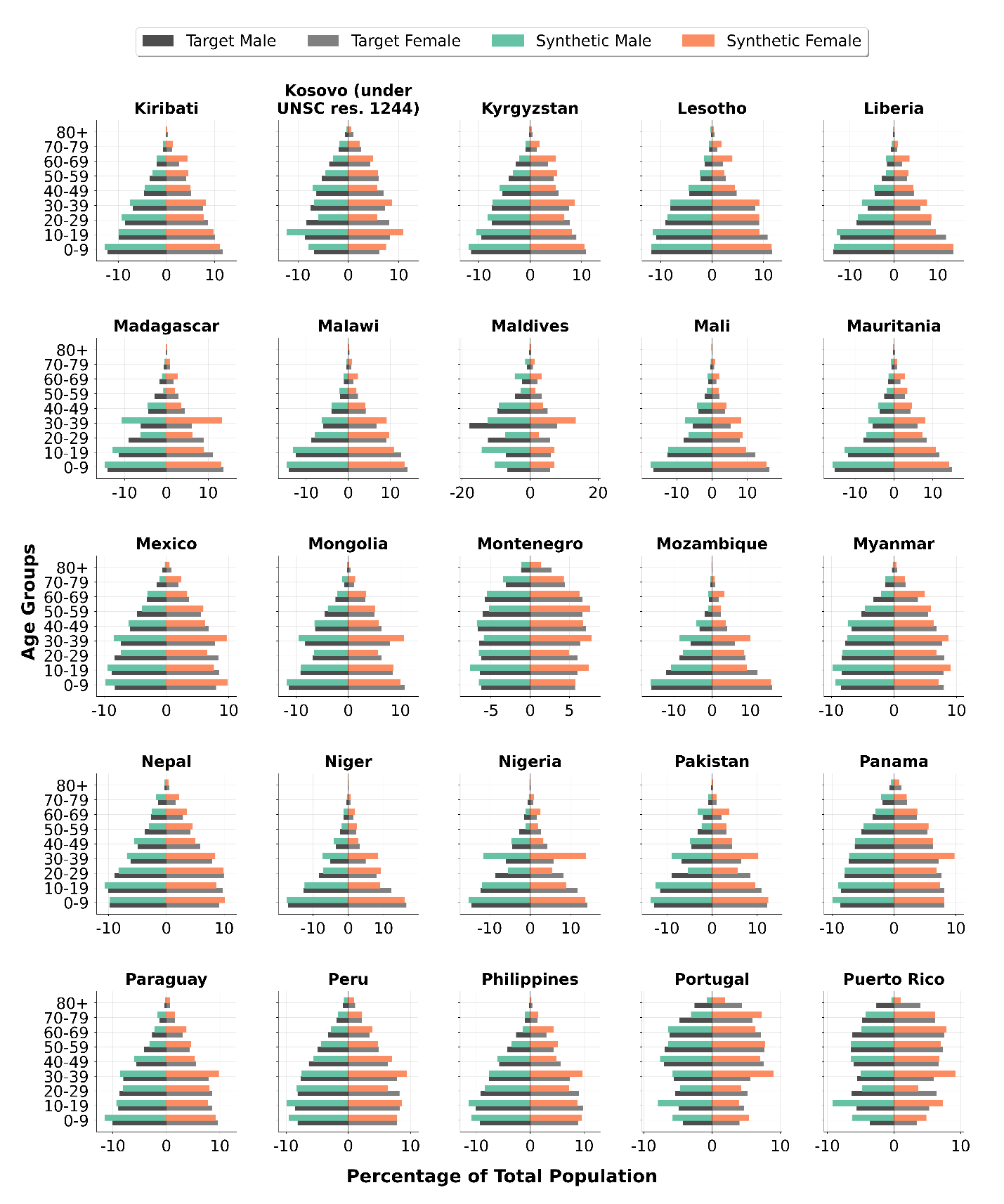
***Fig. D4*** *Synthetic and target age-gender population pyramids for countries K-P*

#

***Fig. D5*** *Synthetic and target age-gender population pyramids for countries Q-T*

#
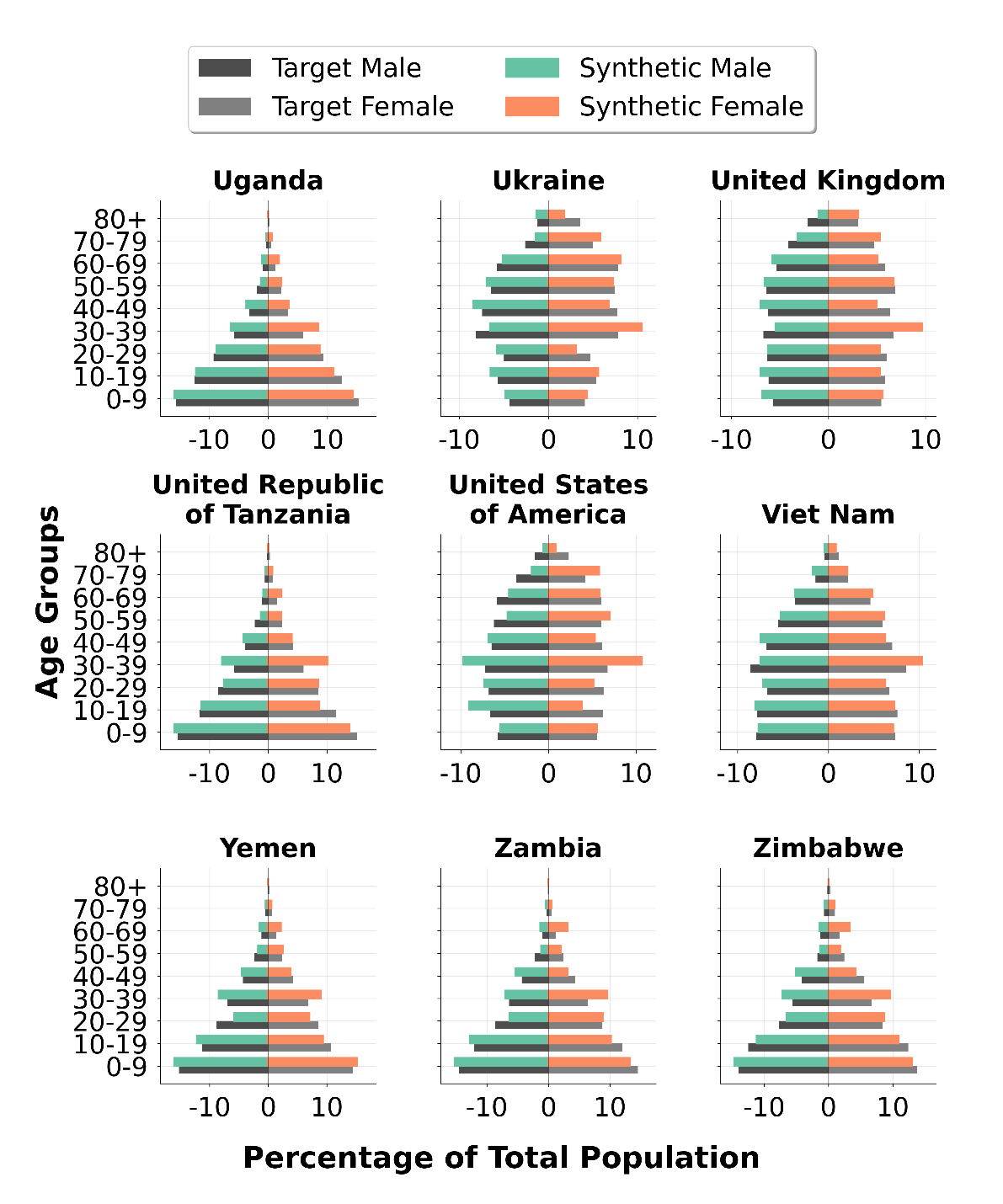


# ***Fig. D6*** *Synthetic and target age-gender population pyramids for countries U-Z*

# Appendix E

Univariate OLS regressions were conducted to examine whether country-level characteristics could explain variation in mean SRMSE across 109 countries. Four predictors were tested: data recency, percentage of unknown values in household composition data, population dependency ratio, and international migrant stock. Scatter plots for each predictor are shown in Figures E1-E4.


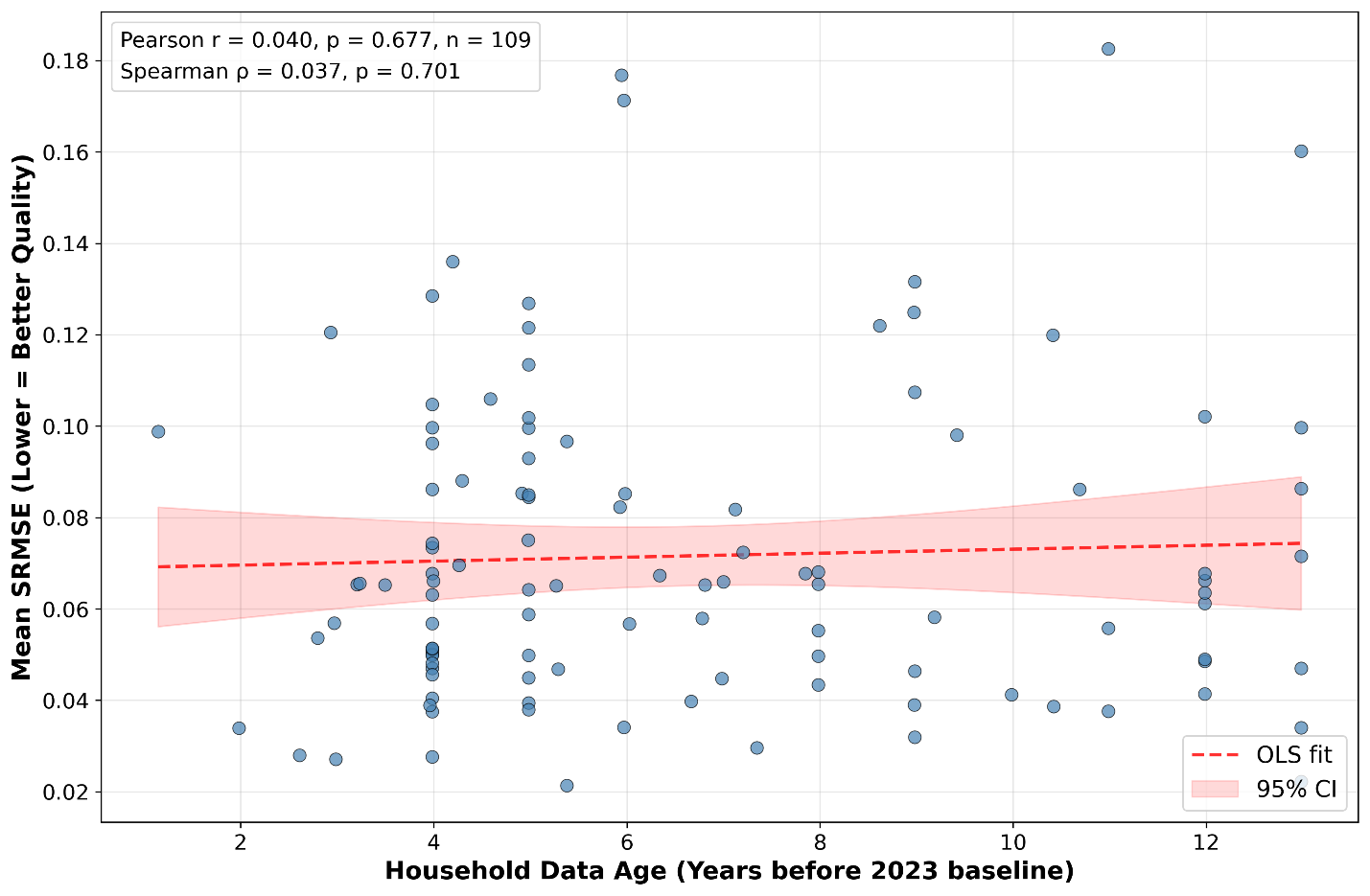
**Fig. E1** Mean SRMSE versus data recency (years before 2023 baseline) across 109 countries.


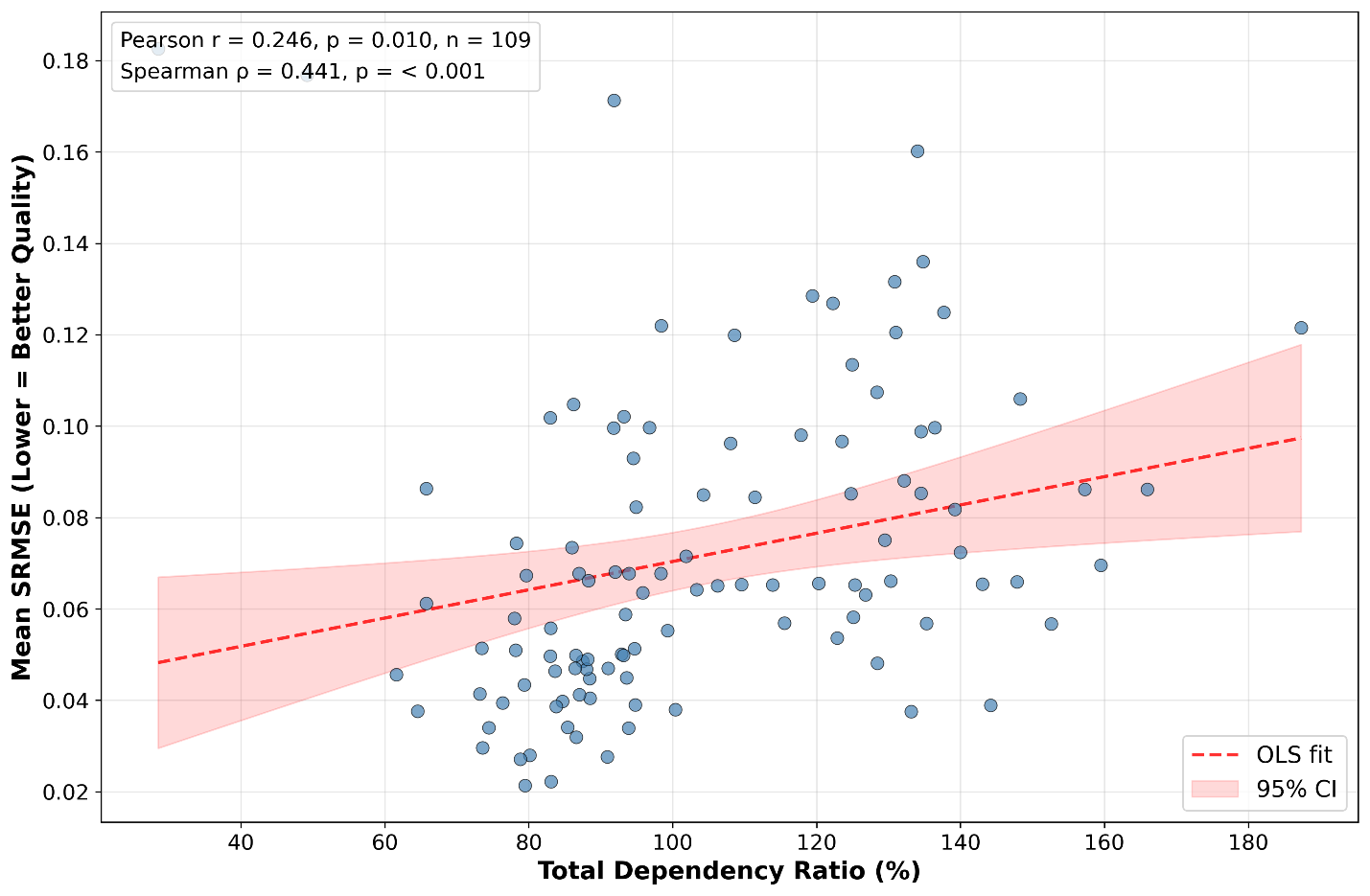
**Fig. E2** Mean SRMSE versus dependency ratio across 109 countries. Dependency ratio is approximated as the number of people aged under 20 and over 60 divided by the number of people aged 20-59

#
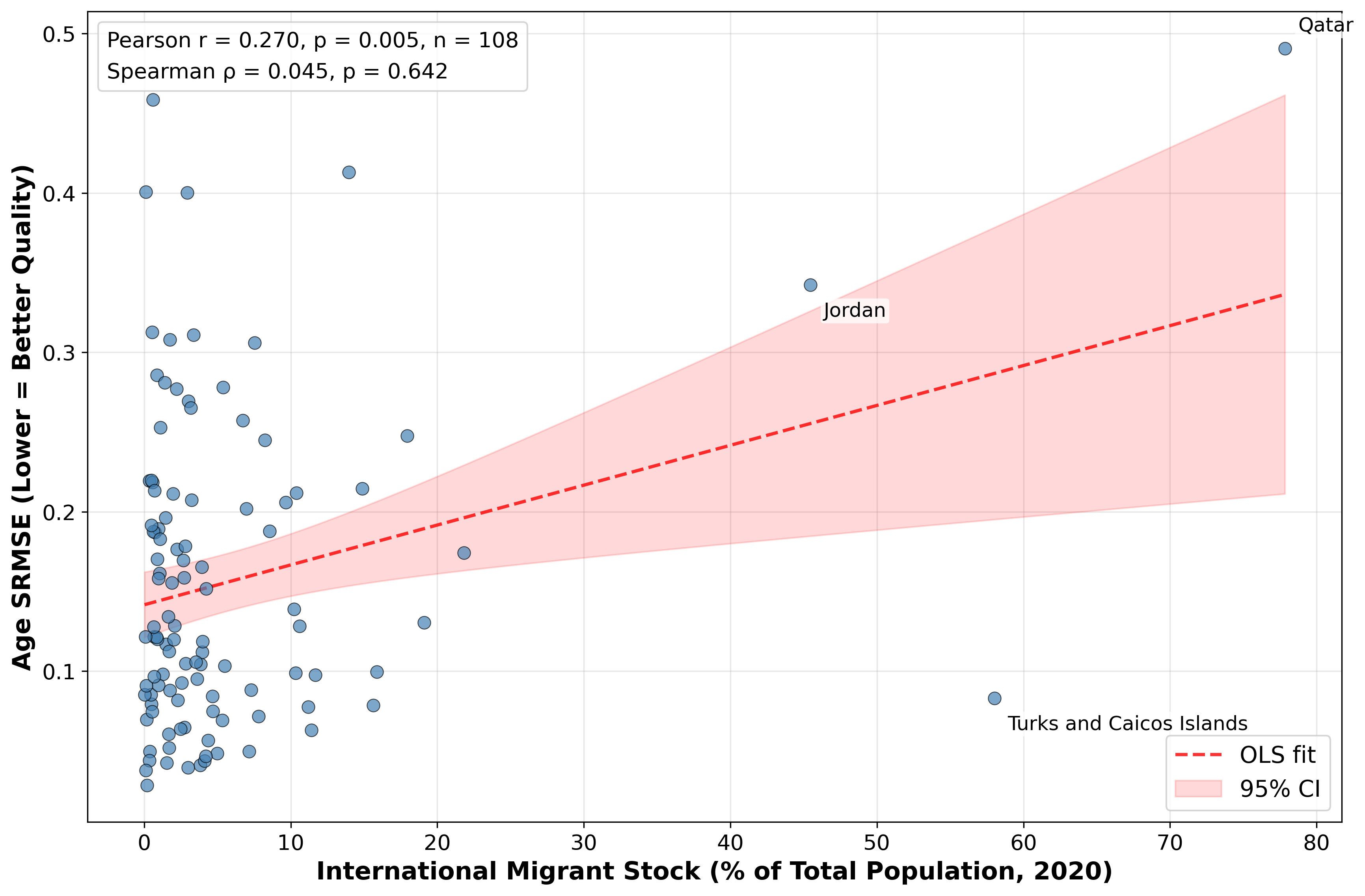


**Fig. E3** Mean SRMSE versus international migrant stock [60] across 109 countries.


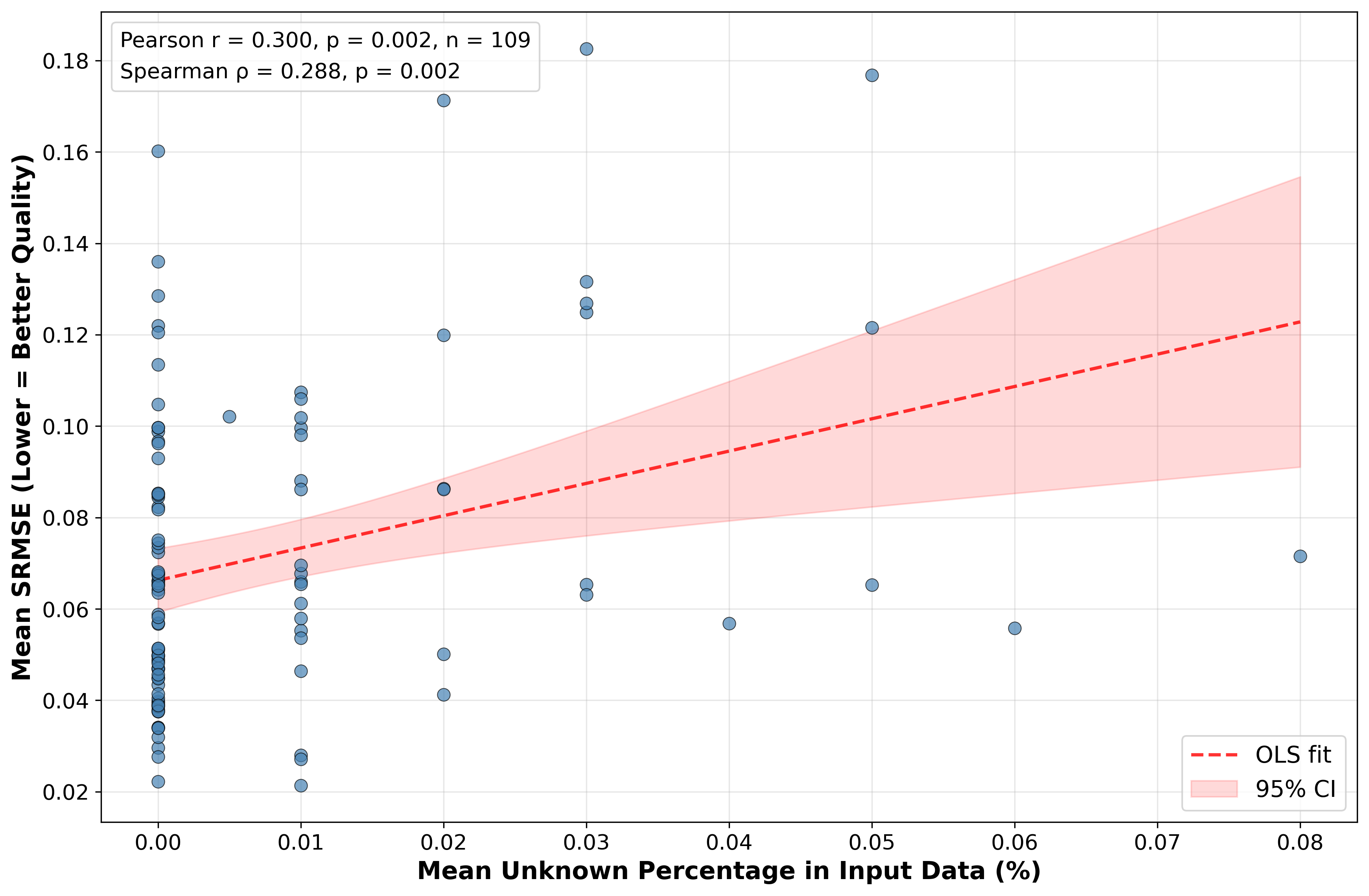
**Fig. E4** Mean SRMSE versus percentage of unknown values in household composition data across 109 countries.

# Appendix F

You are an expert prompt engineer.

# TASK:

Given the following base prompt, generate 10 distinct variants of this prompt, which can be used to test output sensitivity to minor differences in wording.

Adhere to best-practice guidelines, such as:

1. Start with clear instructions

2. Repeat instructions at the end

3. Prime the output

4. Add clear syntax

5. Break the task down

6. State the required JSON output schema, including both required keys and valid values.

Return the prompt variants in plain text format.

# BASE PROMPT:

TASK:

You are an AI assistant generating a realistic synthetic population for {LOCATION}.

Your goal is to add one new household to a growing dataset, so that the generated population matches target distributions.

INSTRUCTIONS:

1. Review the current and target distributions for household size, composition, age, and gender (provided below).

2. Select a household size category where the current count is below its target.

3. Choose a valid household composition for the selected size, prioritizing categories that are currently under-represented.

- 1-person: contains a single adult of any age.

- 2-person: can be either a couple, a lone parent with a child, or unrelated housemates.

- 3+ person: can be a family (couples or lone parents with children, extended family), or unrelated housemates.

- Non-dependent children are aged 18 or older.

4. Select individuals for the household, prioritizing under-represented age groups and genders.

5. For each person, include:

- age (0–120)

- gender ("Male" or "Female")

- relationship (first must be "Head" and be an adult; valid values: "Spouse", "Partner", "Child", "Parent", "Sibling", "Grandchild", "Grandparent", "Housemate", "Lodger", "Aunt", "Uncle", "Nephew", "Niece", "Cousin", "Child-in-law", "Parent-in-law", "Sibling-in-law")

6. Output only a JSON object with a "household" array containing these individuals. Do not include markdown, formatting, or explanations.

DATA:

Household Size Distribution:

{HOUSEHOLD_SIZE_STATS}

Household Composition Distribution:

{HOUSEHOLD_COMPOSITION_STATS}

Age Distribution:

{AGE_STATS}

Gender Distribution:

{GENDER_STATS}

GENERATED HOUSEHOLD:
