# Supplementary material for: A large language model framework for sample-free population synthesis
Source: PLoS One. 2026 Jun 2;21(6):e0341704. doi: 10.1371/journal.pone.0341704 (PMC13229344; doi:10.1371/journal.pone.0341704)
Supplement: Attachment — Submitted filename: PONE-D-26-01425_Reviewer Comments.docx [file pone.0341704.s002.docx]

The manuscript presents a model‑agnostic and sample‑free LLM framework for generating household‑structured synthetic populations using only aggregate demographic data. After benchmarking several models, the authors select GPT‑4o and demonstrate strong performance on simple marginals across 109 countries and in two detailed case studies (Newcastle and Dar es Salaam). More complex attributes show moderate accuracy. The study addresses an important methodological gap, particularly for data‑scarce environments, and offers potential value for applied epidemiological and clinical research. The literature review on synthetic data generation is timely and contributes to an underrepresented but widely used area of research.

**Statistical and Methodological Considerations**

1. *Optimization and Feedback Mechanism*
   The optimization setup in Eq. (1) frames the task as minimizing discrepancies such as SRMSE and JSD. However, its relationship to the iterative prompting procedure is indirect. Because the LLM receives only summarized discrepancies, there is no assurance of consistent improvement or convergence. An ablation comparing generation with and without discrepancy feedback would clarify the role of the feedback loop.
2. *Fit Metrics*
   SRMSE is an appropriate primary metric, but additional measures such as category‑weighted SRMSE or the Wasserstein distance for ordered variables (for example age) would strengthen the evaluation by reducing the influence of small categories and capturing ordering information.
3. *Dependence on LLM Pretraining Data*
   Although the method does not rely on sample data, the demographic relationships produced by the LLM reflect its pretraining data. This may introduce geographic or cultural biases, particularly in regions with limited representation in the training corpus or with atypical demographic structures. A brief discussion of potential future solutions would enhance the manuscript.
4. *Handling of Inconsistent Input Marginals*
   When input marginals originate from inconsistent or heterogeneous sources, as in Dar es Salaam, the LLM reconciles these conflicts implicitly. Without explicit weighting or error modeling, these adjustments may appear arbitrary. A defined conflict‑resolution policy would provide greater transparency.
5. *Lack of Higher‑Order Constraints*
   The framework matches marginal distributions but does not enforce higher‑order joint relationships. Although some joint patterns appear reasonable, the absence of multivariate constraints may lead to artifacts, including unrealistic partner age gaps or household structures.
6. *Reproducibility*
   Reproducibility depends on access to specific model versions, prompts, decoding parameters, and API behavior, which may change over time. The study would benefit from a fully scripted and version‑controlled pipeline.
7. *Validation of Multivariate Structure*
   The manuscript suggests that LLMs guided only by marginals and feasibility rules can recover realistic multivariate dependencies. The Newcastle cross‑tab results support this, but stronger evidence would require quantifying uncertainty and validating held‑out relationships. Bootstrapping across multiple runs and evaluating held‑out descriptors would demonstrate stability and generalization.
8. *Intra‑Household Age Patterns*
   The intra‑household age relationships show general plausibility but also include artifacts such as a bimodal partner age gap and underrepresentation of same‑age couples. These issues appear related to prompt design and the absence of targeted feedback on relational attributes. Incorporating soft priors for key relationships may improve performance.
9. *Model Benchmarking*
   The benchmarking in Table 3 is helpful but incomplete for a methodological paper. Reporting failure modes, decoding settings, prompt lengths, and tokenization effects would provide a more comprehensive comparison. Reproducible scripts for re‑benchmarking would support long‑term validity.
10. *Transparency of Prompts*
    Appendix A provides one example, but the exact prompts used in all analyses (global, Newcastle, Dar es Salaam) should be disclosed. Archiving versioned prompts, if possible, would increase transparency.
11. *Determinants of Global Performance*
    The global evaluation summarizes SRMSE distributions and maps performance across countries. A regression of SRMSE on data quality indicators (such as year of source, percentage unknown, and category counts) and demographic characteristics (such as dependency ratios or migration proxies) would support the claim that data quality and unusual demographic profiles drive errors.
12. *Conflict Reconciliation in Dar es Salaam*
    The framework converges to intermediate values when marginals conflict, which is reasonable. However, this behavior indicates the need for an explicit reconciliation strategy, possibly involving weighting based on survey precision or recency.
13. *Privacy and Re‑identification Risk*
    Although the approach is sample‑free, LLMs may reproduce memorized or rare patterns. The study would be strengthened by:
    a. quantifying the uniqueness or distance of synthetic records relative to available microdata,
    b. checking for potential verbatim leakage when prompts include specific geographic identifiers, and
       c. including an ethics statement covering LLM safety and guardrails.
14. *Comparison With Established Methods*
    A stronger methodological contribution would include direct comparisons with established synthetic population methods, such as GenSynthPop, IPF or IPU, and Bayesian networks, using identical metrics for Newcastle and Dar es Salaam.
15. *Cost and Scalability Analysis*
    The manuscript notes linear cost scaling and provides a cost estimate for the UK. Including empirical measurements of throughput by batch size, experiments with multi‑household generation per call, and evaluation of cache‑aware prompting would clarify the trade‑offs between cost and performance.

**Writing enhancements**

16. The manuscript uses “sex” and “gender” interchangeably; adopting consistent terminology throughout, including in figures, tables, and prompts, would improve clarity.
17. The “other” household category is heterogeneous, providing a brief taxonomy or mapping would improve clarity for readers.
