# Supplementary material for: A large language model framework for sample-free population synthesis
Source: PLoS One. 2026 Jun 2;21(6):e0341704. doi: 10.1371/journal.pone.0341704 (PMC13229344; doi:10.1371/journal.pone.0341704)
Supplement: Attachment — Submitted filename: response_to_reviewers.docx [file pone.0341704.s004.docx]

Dear Dr Mohammad Salah,

We are very grateful for the detailed and thoughtful suggestions from the reviewers and yourself. Whilst most have helped us improve the clarity of the paper, several of the comments required additional simulations and analysis. We have undertaken this work and feel the paper is much stronger as a result. We respond point by point to explain how we have addressed each comment. We hope you feel the revised version now merits publication in PLOS ONE.

Please do not hesitate to contact me if I can be of further assistance.

Yours faithfully

Michael Jones (on behalf of all co-authors)

**Editor**

**1. Please ensure that your manuscript meets PLOS ONE's style requirements, including those for file naming.**

**2. All author-generated code to be made available without restrictions upon publication of the work.**

Both repositories are publicly available without restriction. Links are provided in Section 1 of the manuscript: the population generation library at <https://github.com/MJones235/LLM-Population-Generator/releases/tag/v1.0.0> and the data collection and processing scripts at <https://github.com/MJones235/Synthetic-Population-Experiments/releases/tag/v1.0.0>[.](https://github.com/MJones235/Synthetic-Population-Experiments)

**3. We strongly recommend all authors decide on a data sharing plan before acceptance.**

A data repository has been established at data.ncl.ac.uk and assigned a DOI: <https://doi.org/10.25405/data.ncl.31830205>. The repository contains processed marginals, all prompts used across the global, Newcastle, and Dar es Salaam analyses, configuration files, and example outputs. The repository is currently under review for publication, so the DOI is not yet active. In the interim, it can be viewed using the following private link: <https://figshare.com/s/9a0e2585833c9f75e0ad>.

**4. We note that Figure 1 your submission contain [map/satellite] images which may be copyrighted.**

Figure 1 does not use satellite imagery. All maps are generated programmatically using Plotly. Country boundaries are obtained from Natural Earth, which releases all data into the public domain.

**5. If the reviewer comments include a recommendation to cite specific previously published works, please review and evaluate these publications to determine whether they are relevant and should be cited**

Not applicable

**Reviewer 1**

**Abstract:**
**Line 9 and 10: is it the traditional synthesis methods that heavily rely on micro data? If yes, then you could contextualize it other than leaving it open. What makes micro data unavailable? Is it restrictions due to privacy? May be you could add it’s not being available in many cases, the restriction due to privacy or being obsolete.**

The abstract has been revised to clarify that it is the established synthesis methods that are dependent on microdata, and to contextualise why microdata are often unavailable: they are infrequently collected on a decadal cycle, restricted for privacy protection, and typically released only as small public-use samples at coarse geographic scales.

**Line 15: Criteria for training the LLM is not visible. Which kind of data was used in the pre-training stage? Other than saying the model “draws on prior knowledge encoded in its training to propose plausible attribute combinations”, could you include how the training was done?**

The abstract has been updated to use "pre-training" rather than "training" to avoid implying that the LLM was fine-tuned for this task. Section 3.6 has been revised to clarify that the framework uses pre-trained foundation models without modification. This is a deliberate design choice to minimise data requirements and ensure the framework is accessible to practitioners.

**Lines 20 and 21: besides results from the different studies demonstrating applicability of the framework in data rich and scarce environments, what are the major conclusions and recommendations you draw from testing the model.**

The abstract has been revised to state explicitly that the principal contribution of the framework is to enable population synthesis in data-constrained settings, expanding the applicability of agent-based modelling.

**Background**
**Line 96: how about if “….. introduced an iterative process that builds households one member at a time” is rewritten as “….. introduced an iterative process that builds households member by member”.**

Revised as suggested.

**Methods**
**Line 180: crosstabulation to cross tabulation or cross-tabulation.**

Revised to "cross-tabulation" throughout the manuscript.

**Line 279 to 282: What are the 3 dimensions for evaluating outputs? If they are distributional fit to the target descriptors, structural feasibility of the generated records, and computational efficiency in terms of runtime and token cost then what is statistical accuracy, generation speed and affordability? Do they mean the same things? Could it be possible to be consistent and also add parameters in table 3 here to show which one belongs to which dimension.**

The evaluation framework has been revised to use three consistent dimensions throughout: (i) distributional fit, (ii) structural feasibility, and (iii) computational efficiency. The paragraph introducing Table 4 has been updated to map each column explicitly to its corresponding dimension.

**Results**
**Lines 331 to 334: GPT-40 was selected as the best model overall but GRO-40 mini scored better in total cost, time and they both tied in success rate. For a limited resource setting one could opt for GRO-40 mini even with the lower accuracy. Could it be possible to further justify the selection of GRO-40 other than just saying it demonstrated the best balance between distributional fit, speed and reliability. If there is any kind of weight attached to the different parameters of performance measures may be it would be better.**

The selection of GPT-4o over GPT-4o-mini is further justified in Appendix C, which shows the age distribution produced by each model. The difference in JSD translates to a visually noticeable degradation in distributional quality for GPT-4o-mini. While GPT-4o-mini achieved lower cost, its distributional fit was inferior to GPT-4o, making it unsuitable where accuracy is the primary criterion.

*Fig. C1 Age-gender population pyramids for all benchmarked models, based on an 800-household test case.*

**Line 331, and 410: says “For Newcastle and Dar es Salaam, larger populations of approximately 100,000 individuals were generated to enable detailed evaluation of internal structure and feasibility”. Table 3 measures performance on a 500 household test case. Why use 500 households for model selection yet test cases had 100,000 individuals and convergence reached at about 800 households.**
**Just out of curiosity, do the results in table 3 remain the same if you use 800 households when convergence occurs to measure LLM performance?**

The benchmarking experiment was repeated using 800 households, consistent with the convergence point identified in Section 5.3. Relative model performance was largely unchanged. Section 3.6 and Table 4 have been updated accordingly.

**Discussion**
**Line 447: double space between demographic and characteristics; reduce to single space.**

Done

**Limitations**

**Line 538 to 543: it is great to note that the internal thinking process of LLM is not inspectable which complicates consistency, reproducibility and auditability.**

Thank you - no change required

**In line 99 to 100 which says, “sample free models become increasingly complex as more attributes are added stated” could this be part of the limitations? If yes, add it to the limitations and how it was handled.**

This point is addressed in Section 6.7 as a direction for future work rather than a confirmed limitation. The study focuses on a limited set of demographic variables, and while other methods are known to struggle with high-dimensional attribute spaces, this has not yet been established as a limitation of the LLM-based approach. Further investigation is left as a suggestion for future work.

**Conclusion**
**Line 566 to 568: how about results with SRME are kept in the results section and we have conclusions as typical conclusions.**

The conclusion has been revised to avoid reporting specific numerical results, instead summarising findings in qualitative terms.

**Line 568 to 568: how about you revised “The framework was shown to provide viable populations even when input data were sparse or inconsistent” to “The framework provided viable populations even when input data were sparse or inconsistent.”. This uses active voice and makes it more direct.**

Revised as suggested.

**Reviewer 2**

**Statistical and Methodological Considerations**
**1. Optimization and Feedback Mechanism**
**The optimization setup in Eq. (1) frames the task as minimizing discrepancies such as SRMSE and JSD. However, its relationship to the iterative prompting procedure is indirect. Because the LLM receives only summarized discrepancies, there is no assurance of consistent improvement or convergence. An ablation comparing generation with and without discrepancy feedback would clarify the role of the feedback loop.**

A preliminary experiment was conducted in which households were generated without distributional feedback — the prompt contained the target distributions but no information about the population generated so far. As shown in Figure D1 (Appendix D), distributional fit was poor under this configuration: household composition was dominated by "couple with dependent children" households (>60% of generated records against a 17% target), household size significantly overrepresented 3–4 persons, and the age distribution collapsed to working-age adults and children. These results confirm that the iterative feedback mechanism is essential to achieving distributional alignment, and that supplying target distributions within the prompt alone is insufficient. The justification for this design choice has been placed in Section 3.4, where the feedback algorithm is described, and full details of the preliminary experiment are provided in Appendix D.

*Fig. D1 Marginal distributions produced without distributional feedback, compared against Newcastle upon Tyne targets.*

**2. Fit Metrics**
**SRMSE is an appropriate primary metric, but additional measures such as category weighted SRMSE or the Wasserstein distance for ordered variables (for example age) would strengthen the evaluation by reducing the influence of small categories and capturing ordering information.**

The relationship between SRMSE and Wasserstein distance for age across 109 countries is discussed in Section 5.2.1 and illustrated in Figure E1, Appendix E. The two metrics diverge for a subset of countries, with the direction of divergence related to the shape of the population pyramid. Countries with expansive pyramids, such as Nigeria, Madagascar and Sudan, tend to have high SRMSE relative to their Wasserstein distance, as category-level errors in these distributions are penalised more heavily by SRMSE. The opposite pattern is observed for countries with constrictive pyramids, such as Italy, Portugal and Canada. The implications of this metric sensitivity are discussed in Section 6.2: Africa appears to underperform relative to other continents under SRMSE, but this difference is considerably reduced when Wasserstein distance is used instead. Which metric better reflects fitness for purpose will depend on the intended application.

**3. Dependence on LLM Pretraining Data**
**Although the method does not rely on sample data, the demographic relationships produced by the LLM reflect its pretraining data. This may introduce geographic or cultural biases, particularly in regions with limited representation in the training corpus or with atypical demographic structures. A brief discussion of potential future solutions would enhance the manuscript.**

The potential for geographic and cultural bias introduced by the LLM's pre-training data is discussed in Section 6.2, where performance is evaluated across demographic regimes and regions. Two mitigation strategies are proposed in Section 6.7. First, prompt augmentation could improve the model's demographic priors for underrepresented regions, with regional context supplied either manually or retrieved automatically from publicly available sources such as Demographic Health Surveys or UN statistics. Second, the synthetic population could serve as pseudo-microdata for a subsequent IPU reweighting step, allowing classical fitting techniques to correct residual distributional errors after generation. The effectiveness of this hybrid approach would depend on the LLM having generated a sufficiently diverse pool of household structures for the reweighting to act upon.

**4. Handling of Inconsistent Input Marginals**
**When input marginals originate from inconsistent or heterogeneous sources, as in Dar es Salaam, the LLM reconciles these conflicts implicitly. Without explicit weighting or error modeling, these adjustments may appear arbitrary. A defined conflict resolution policy would provide greater transparency.**

Two prompt configurations were tested for Dar es Salaam: a baseline (E1) presenting all marginals without prioritisation or conflict resolution guidance, and a modified version (E2) in which explicit instructions were added to prioritise household size alignment above other marginals. Contrary to expectation, mean household size was identical under both configurations (3.87 persons in each case), suggesting that the added instructions did not alter the model's sampling behaviour for this variable. Fit to the remaining distributions deteriorated under E2, with mean SRMSE increasing from 0.107 to 0.159.

These results suggest that conflict resolution is better handled as a preprocessing step prior to generation, where targets can be explicitly reconciled before being passed to the model, rather than through in-prompt instructions that require the LLM to simultaneously interpret inconsistent targets and generate plausible households. This analysis (and consequently the text for Prompt 2) was not included in the main manuscript as the experiment was inconclusive with respect to the original question, and a more systematic prompt comparison would be needed to draw firm conclusions. The baseline configuration E1 was retained for all subsequent analyses and is reproduced in Appendix A.

Prompt E2 is reproduced below:

INSTRUCTIONS:

1. Review the current and target distributions for household size, age, and gender (provided below).

2. Select a household size.

- Alignment to the target mean is the top priority

- Prefer under-represented sizes

3. Choose household composition after size is fixed. Fitting this distribution is lower priority.

- 1 person: contains a single adult of any age.

- 2 person: can be either a couple, a lone parent with a child, or unrelated housemates.

- 3+ person: can be a family (couples or lone parents with children, extended family), or unrelated housemates.

4. Select individuals for the household, prioritizing under-represented age groups and genders.

5. For each person, include:

- age (0–120)

- gender ("Male" or "Female")

- relationship (first must be "Head" and be an adult; valid values: "Spouse", "Partner", "Child", "Parent", "Sibling", "Grandchild", "Grandparent", "Housemate", "Lodger", "Aunt", "Uncle", "Nephew", "Niece", "Cousin", "Child-in-law", "Parent-in-law", "Sibling-in-law")

6. Output only a JSON object with a "household" array containing these individuals. Do not include markdown, formatting, or explanations.

**5. Lack of Higher Order Constraints**
**The framework matches marginal distributions but does not enforce higher order joint relationships. Although some joint patterns appear reasonable, the absence of multivariate constraints may lead to artifacts, including unrealistic partner age gaps or household structures.**

A dedicated paragraph has been added to Section 6.6 acknowledging that the framework does not enforce higher-order joint constraints during generation. Although some multivariate relationships are recovered implicitly, this is not guaranteed. Specific relational artifacts documented in Section 5.3, including a bimodal partner age gap and underrepresentation of same-age couples, are noted as likely reflecting the absence of targeted relational feedback in the prompt.

**6. Reproducibility**
**Reproducibility depends on access to specific model versions, prompts, decoding parameters, and API behavior, which may change over time. The study would benefit from a fully scripted and version controlled pipeline.**

All code is available in version-controlled public repositories, with links provided in the introduction of the manuscript. The exact model snapshots used in each experiment are documented in Table 4, and the codebase includes scripts for testing the framework with alternative model versions. A note on reproducibility has been added to Section 6.6 acknowledging that access to specific model versions cannot be guaranteed as APIs evolve, and directing readers to the documented workflow for substituting alternative versions.

**7. Validation of Multivariate Structure**
**The manuscript suggests that LLMs guided only by marginals and feasibility rules can recover realistic multivariate dependencies. The Newcastle cross tab results support this, but stronger evidence would require quantifying uncertainty and validating held out relationships. Bootstrapping across multiple runs and evaluating held out descriptors would demonstrate stability and generalization.**

A bootstrap resampling analysis was conducted across 20 repeated runs of 800 households to quantify uncertainty in the R² values for multivariate cross-tabulations (Table 7). $R^{2}$ values were broadly stable across runs, with confidence intervals tightest for tightest for household composition by size ($R^{2}$= 0.666, 95% CI = [0.646, 0.695]) and widest for household composition by gender ($R^{2}$= 0.778, 95% CI = [0.656, 0.894]).

**8. Intra Household Age Patterns**
**The intra household age relationships show general plausibility but also include artifacts such as a bimodal partner age gap and underrepresentation of same age couples. These issues appear related to prompt design and the absence of targeted feedback on relational attributes. Incorporating soft priors for key relationships may improve performance.**

The artifacts identified in Section 5.3, including the bimodal partner age gap and underrepresentation of same-age couples, are acknowledged in the limitations (Section 6.6) as likely reflecting the absence of targeted relational feedback in the prompt. The suggestion to incorporate soft priors for key relationships is well taken and noted as a direction for future prompt development. We agree that targeted relational constraints, if incorporated into the prompt design or validation loop, could reduce these artifacts, and this is noted as a direction for future work in Section 6.7.

**9. Model Benchmarking**
**The benchmarking in Table 3 is helpful but incomplete for a methodological paper. Reporting failure modes, decoding settings, prompt lengths, and tokenization effects would provide a more comprehensive comparison. Reproducible scripts for re benchmarking would support long term validity.**

Failure modes have been added to Table 4, reporting counts of JSON errors, schema errors, API errors, and failed households for each model. Three additional domain-specific validation rules were applied — each household must contain exactly one head, no minors may live alone, and parents must be older than children — and no violations were observed for any model. Sampling parameters (temperature = 1.0, top-p = 1.0) are reported in Section 5.1. Token counts and cost calculations are reported in full in Table 4, including mean input and output tokens per prompt, total tokens, and cost per household. Differences in mean input token counts reflect re-prompting overhead for models with higher failure rates, and differences in tokenisation strategy across model families. All scripts are available in the public repositories listed in Section 1.

**10. Transparency of Prompts**
**Appendix A provides one example, but the exact prompts used in all analyses (global, Newcastle, Dar es Salaam) should be disclosed. Archiving versioned prompts, if possible, would increase transparency.**

All prompts used in the global, Newcastle, and Dar es Salaam analyses are now archived in the data repository. Appendix A retains an illustrative example for readability; the full versioned prompt set is available in the repository.

**11. Determinants of Global Performance**
**The global evaluation summarizes SRMSE distributions and maps performance across countries. A regression of SRMSE on data quality indicators (such as year of source, percentage unknown, and category counts) and demographic characteristics (such as dependency ratios or migration proxies) would support the claim that data quality and unusual demographic profiles drive errors.**

Univariate OLS regressions were conducted to examine whether four candidate country-level predictors (data recency, percentage of unknown values in household data, population dependency ratio, and international migrant stock) could explain variation in mean SRMSE across 109 countries. Results are reported in full in Appendix F.

None of the predictors showed a strong association with SRMSE. Data recency showed no relationship (Pearson r = 0.040, p = 0.677). The percentage of unknown values and the dependency ratio each showed weak but statistically significant Pearson correlations (r = 0.300 and r = 0.246 respectively), but each explained less than 10% of variance in SRMSE. For international migrant stock, the Pearson correlation was significant (r = 0.295, p = 0.002) but the Spearman rank correlation was not (ρ = 0.042, p = 0.667), suggesting the result is driven by a small number of high-migration outliers rather than a general trend.

In light of this, the discussion in Section 6.2 has been revised to be clearer. The original assertion that performance is shaped by three identifiable factors has been reframed. We now propose that data quality, demographic representation in the model's training data, and household category definition, are plausible hypotheses for model performance, but are no longer presented as established conclusions. The regression results are reported in Section 6.2 with reference to Appendix E and the discussion distinguishes clearly between what was observed and what remains hypothetical. The analysis also notes that where input marginals are contradictory, as in Dar es Salaam, a perfect fit is logically impossible regardless of model quality, and that the observed relationship between category definition and composition fit is suggestive but based on limited configurations. Disentangling these effects would require a dedicated experimental study with controlled variation in input conditions and is noted in the Discussion section as a direction for future work.

**12. Conflict Reconciliation in Dar es Salaam**
**The framework converges to intermediate values when marginals conflict, which is reasonable. However, this behavior indicates the need for an explicit reconciliation strategy, possibly involving weighting based on survey precision or recency.**

This comment overlaps substantially with Comment 4, which also concerns conflict resolution in Dar es Salaam. As described in the response to Comment 4, two prompt configurations were tested: a baseline presenting all marginals without prioritisation, and a modified version with explicit conflict resolution instructions. The modified prompt did not improve alignment with the prioritised target and reduced fit to other distributions, suggesting that explicit in-prompt reconciliation is not straightforward. The baseline behaviour — converging to an intermediate value that partially satisfies competing constraints — is documented in Section 5.4. We agree that a more principled reconciliation strategy, applied as a preprocessing step prior to generation, would be a valuable direction for future work, and this is noted in Section 6.7.

**13. Privacy and Re identification Risk**
**Although the approach is sample free, LLMs may reproduce memorized or rare patterns. The study would be strengthened by:**
**a. quantifying the uniqueness or distance of synthetic records relative to available microdata,**
**b. checking for potential verbatim leakage when prompts include specific geographic identifiers, and**
**c. including an ethics statement covering LLM safety and guardrails.**

An ethics statement has been added addressing all three sub-points raised.

Regarding re-identification risk: the synthetic records contain no quasi-identifiers, such as names, addresses, dates of birth, or geographic identifiers below city level, and therefore cannot be linked to real individuals. The safeguarded microdata used in the IPU comparison is anonymised through broad age banding and geographic restriction for the same reason; apparent similarity between synthetic and real records does not therefore constitute a privacy breach.

Regarding verbatim leakage: this risk applies primarily to free-form text generation. The outputs of this framework are short, structured JSON records drawn from a fixed categorical schema, making verbatim reproduction of training data highly implausible.

Regarding LLM safety and guardrails: LLMs were accessed via two routes. Commercial APIs through Azure AI Foundry operate under published usage policies with content filtering and safety guardrails. Locally hosted models were accessed via Ollama. The framework did not fine-tune or otherwise modify the underlying models, and prompts contained only aggregate statistical targets. No content was generated beyond structured demographic records within a fixed categorical schema.

**14. Comparison With Established Methods**
**A stronger methodological contribution would include direct comparisons with established synthetic population methods, such as GenSynthPop, IPF or IPU, and Bayesian networks, using identical metrics for Newcastle and Dar es Salaam.**

A direct comparison with IPU has been added as Section 5.5, using LSOA E01008291 in Newcastle upon Tyne (883 households) as the test case. Twenty independent populations were generated by each method to assess stability. The LLM framework achieved better distributional fit for age (SRMSE: 0.013 vs 0.058), gender (0.003 vs 0.011), and household size (0.064 vs 0.074). IPU achieved substantially better fit for household composition (SRMSE: 0.081 vs 0.206). Mean SRMSE favoured IPU (0.056 vs 0.072), driven by the composition result, while mean Wasserstein distance favoured the LLM framework (0.032 vs 0.065). The overall ranking depends on the choice of metric and the relative weight assigned to each variable.

A comparison was not possible for Dar es Salaam, as IPU requires microdata that are unavailable in that context. Comparisons with GenSynthPop and Bayesian networks were not conducted as these methods similarly require microdata or detailed cross-tabulations as inputs, making them inapplicable in data-scarce settings. This inapplicability is the core motivation for the LLM framework. Where microdata are available, as in Newcastle, IPU or related methods would likely be preferable given their lower computational cost and comparable distributional fit on most variables.

**15. Cost and Scalability Analysis**
**The manuscript notes linear cost scaling and provides a cost estimate for the UK. Including empirical measurements of throughput by batch size, experiments with multi household generation per call, and evaluation of cache aware prompting would clarify the trade offs between cost and performance.**

A systematic batch size experiment has been added as Section 6.5. Batch sizes from 1 to 35 were tested using GPT-4o and the Newcastle case study, with 10 repeated runs per configuration. Results are summarised in Figure 14. Generation time per household and cost followed a U-shaped profile, reaching a minimum at a batch size of around 10. Reliability remained high up to a batch size of 20, beyond which failure rates increased steeply, exceeding 40% at a batch size of 35. Output diversity peaked at batch sizes of 15–25 before declining, coinciding with the onset of elevated failure rates. Distributional fit improved for age and household size as batch size increased from 1 to 10, while household composition fit degraded with increasing batch size. A batch size of 10 was identified as the optimal configuration, reducing generation time by 59%, cost by 70%, and API call volume by 90% compared to the single-household default, while improving mean SRMSE from 0.133 to 0.089. Cache-aware prompting was not evaluated in this study, as the dynamic nature of the prompt, which updates the current population statistics at each iteration, means that caching would not be applicable in the standard generation workflow.

**Writing enhancements**
**16. The manuscript uses “sex” and “gender” interchangeably; adopting consistent terminology throughout, including in figures, tables, and prompts, would improve clarity.**

Terminology has been standardised to "gender" throughout the manuscript, consistent with the term used in the prompt. This update has been applied to the main text, tables, and figures.

**17. The “other” household category is heterogeneous, providing a brief taxonomy or mapping would improve clarity for readers.**

Table 3 has been added to Section 4, providing a complete mapping of household relationship structures to both the UK and global classification schemes. This clarifies the composition of the "other" category in the UK scheme, which encompasses households containing combinations of relatives not covered by the standard categories, as well as housemate and unrelated combinations.

**Reviewer 3**

**1. Report SDs or 95% CIs for SRMSE across repeated runs, for both the global and Newcastle experiments.**

Standard deviations and summary statistics for SRMSE are reported across repeated runs for both experiments. For the global evaluation, Table 5 reports the mean, median, standard deviation, IQR, minimum and maximum SRMSE across 109 countries for each variable. For the Newcastle experiment, Table 6 reports the same statistics across 20 independent runs of 800 households. Bootstrap confidence intervals for the multivariate R² values reported in Section 5.3 are provided in the bootstrap table within that section.

**2. State exact model snapshots (e.g. GPT-4o-2024-11-20), temperature, and top-p. Section 6.4 shows these matter.**

Exact model version snapshots are reported in Table 4 for all benchmarked models. Sampling parameters were fixed at temperature = 1.0 and top-p = 1.0 across all experiments, including the prompt sensitivity analysis in Section 6.4. These are reported in Section 5.1.

**3. Date the cost figures in Table 3 and add a cost-per-household metric.**

Cost figures in Table 5 (formerly Table 3) are dated as of September 2025. A cost-per-household metric has been added as a column in Table 5.

**4. Finalize the data repository with a DOI before acceptance. List what it contains (processed marginals, prompts, configs, eval scripts, example outputs).**

A data repository has been established at data.ncl.ac.uk and assigned a DOI: <https://doi.org/10.25405/data.ncl.31830205>. The repository contains processed marginals, all prompts used across the global, Newcastle, and Dar es Salaam analyses, configuration files, and example outputs. The repository is currently under review for publication, so the DOI is not yet active. In the interim, it can be viewed using the following private link: <https://figshare.com/s/9a0e2585833c9f75e0ad>.

**5. In Dar es Salaam, household size says 14.5% one-person households while composition says 9.6%, and the model lands on 12.8%. Explain how the feedback loop balances competing targets. Are all marginals weighted equally? This will come up in any real-world application with inconsistent inputs.**

The conflict between household size and composition marginals in Dar es Salaam is discussed in Section 5.4. The generated population contained 12.8% one-person households, intermediate between the two conflicting targets of 14.5% and 9.6%. This suggests that the framework treats competing marginals as approximately equally weighted in the absence of explicit prioritisation instructions, partially satisfying each constraint. As noted in the response to Comment 4 (Reviewer 2), an attempt was made to provide explicit conflict resolution guidance via a modified prompt, but this did not alter the outcome for the prioritised variable. We agree that conflict resolution is an important consideration for real-world applications, and note in Section 6.7 that preprocessing marginals to resolve inconsistencies before generation would be a more reliable approach than in-prompt instructions.

**6. Report the rate of structurally invalid households (e.g. child-aged spouse) before and after the validation step. This would show how much work the LLM does versus the rule checker.**

Table 4 has been updated to include counts of JSON, Schema and API errors. Custom validation rules were also checked (e.g. one head; no children living alone; parents older than children), but there were no violations by any of the models.

**7. Figure 4 (25 pyramids on one page) is hard to read in print. Move some to supplementary.**

Figure 4 has been replaced with a 3×3 grid showing nine countries, each with a larger sub-figure, selected for their geographic and demographic diversity. Population pyramids for all 109 countries in the evaluation set have been added to Appendix E, organised alphabetically across four 5×5 grids and one 3×3 grid. The main text has been updated to refer readers to Appendix E for the full set of results.

**8. Figure 12 has missing bars that the text explains as generation failures. Add this to the caption.**

The caption for Figure 12 (now Figure 13, following renumbering) has been updated to explain that missing bars indicate failed generations, defined as runs in which fewer than 450 of the 500 target households met the feasibility criteria defined in Section 3.5.2.

**9. References 44-45 are both World Population Prospects — combine into one.**

References 44 and 45, both citing World Population Prospects, have been combined into a single reference. In-text citations have been updated accordingly.

**10. Reference 42 (Lim et al.) is still on arXiv. Update if published.**

Reference 42 (Lim et al.) remains on arXiv and has not yet been published in a peer-reviewed journal at the time of resubmission. The arXiv citation has been retained.

**11. Fix “occassion” (p. 23) and standardize hyphenation of “sample-based.”**

The spelling error "occassion" has been corrected to "occasion". A search for non-standard hyphenation of "sample-based" and "sample-free" was conducted throughout the manuscript; both terms are used consistently in their hyphenated forms.

**A note for the discussion: the systematic age and composition biases could matter for downstream transport, health, or disaster simulations. A sentence connecting these to application risks would help practitioners.**

A sentence has been added to Section 6.2 noting that where biases in age distribution or household composition persist, they may have practical consequences for downstream applications in transport, health, and disaster management, where demographic structure directly shapes individual behaviour and vulnerability.
