# Supplementary material for: A large language model framework for sample-free population synthesis
Source: PLoS One. 2026 Jun 2;21(6):e0341704. doi: 10.1371/journal.pone.0341704 (PMC13229344; doi:10.1371/journal.pone.0341704)
Supplement: Attachment — Submitted filename: response-to-reviewers.docx [file pone.0341704.s005.docx]

**Typographical corrections**

A number of typographical errors identified during final proofreading have been corrected:

- "Tables 4" has been corrected to "Table 4" (Section 6.1);
- "along" has been corrected to "alone" in the description of domain-specific validation rules (Section 6.1);
- "addition" has been corrected to "additional" in the caption to Table 2;
- "inference were" has been corrected to "inference times were" (Section 6.1);
- "that reflects" has been corrected to "that reflect" in the list of contributions (Section 1);
- "established" has been corrected to "establishes" in the methodology overview (Section 3.1);
- the confidence interval "[0.465, 0661]" has been corrected to "[0.465, 0.661]" in Table 8;
- a missing comma has been inserted after citation [20,21] in Section 2;
- the cross-reference "Figure 12" in Section 6.4 has been corrected to "Figure 13";
- the double full stop in the caption to Figure E4 has been removed; and,
- "appesar" has been corrected to "appear" in the ethics statement.

**Reference update**

Reference [42] (Lim et al.) has been updated to reflect its accepted publication in *Transportation Research Part C: Emerging Technologies* (2026, vol. 185, 105581; doi:10.1016/j.trc.2026.105581). The reference was previously cited as a preprint; the published version details have now been substituted.

**Data availability statement**

The data availability statement has been updated as the DOI is now live.

The statement reads: "All data and code supporting this study are publicly available. Processed marginals, prompts, configuration files, and example outputs are archived in the Newcastle University data repository (<https://doi.org/10.25405/data.ncl.31830205>). The population generation library is available at <https://github.com/MJones235/LLM-Population-Generator/releases/tag/v1.0.0> and data collection and processing scripts at <https://github.com/MJones235/Synthetic-Population-Experiments/releases/tag/v1.0.0>."
